# Strategies and Timing of Complete Revascularization in STEMI Patients with Multivessel Coronary Artery Disease

**DOI:** 10.3390/jcm15124667

**Published:** 2026-06-16

**Authors:** Domenico Simone Castiello, Claudia Rocca, Letizia Rosa Romano, Carmen Anna Maria Spaccarotella, Alberto Polimeni, Mario Chiatto, Antonio Curcio, Giovanni Esposito, Ciro Indolfi

**Affiliations:** 1 Department of Advanced Biomedical Sciences, Federico II University, 80131 Naples, Italy; ds.castiello@gmail.com (D.S.C.); carmenannamaria.spaccarotella@unina.it (C.A.M.S.); espogiov@unina.it (G.E.); 2 Department of Pharmacy, Health and Nutritional Sciences, University of Calabria, 87036 Rende, Italy; crocca0@gmail.com (C.R.); leti.romano7@gmail.com (L.R.R.); alberto.polimeni@unical.it (A.P.); antonio.curcio.cardio@unical.it (A.C.); 3 Division of Interventional Cardiology, Annunziata Hospital, 87100 Cosenza, Italy; 4 Annunziata Hospital, 87100 Cosenza, Italy; mariochiatto53@gmail.com; 5 Fondazione Italiana Cuore e Circolazione, Italian Society of Cardiology, 00198 Rome, Italy

**Keywords:** acute coronary syndrome (ACS), myocardial infarction (MI), ST-segment elevation myocardial infarction (STEMI), percutaneous coronary intervention (PCI), non-culprit lesions, functional assessment, intracoronary imaging

## Abstract

Multivessel coronary artery disease is observed in a substantial proportion of patients presenting with ST-segment elevation myocardial infarction (STEMI) and identifies a higher-risk phenotype characterized by larger atherosclerotic burden, recurrent ischemic events, and greater need for subsequent revascularization. Over the past decade, randomized evidence has progressively shifted the interventional paradigm from culprit-lesion-only primary percutaneous coronary intervention (PCI) toward complete revascularization in hemodynamically stable STEMI patients with suitable non-culprit lesions. Nevertheless, several clinically relevant questions remain unresolved, including the optimal criteria for selecting non-culprit lesions, the relative value of angiography, coronary physiology, and intracoronary imaging, the timing of complete revascularization, and the management of patients presenting with cardiogenic shock. Angiography-guided complete revascularization has the strongest evidence base, while physiology-guided approaches may reduce unnecessary PCI but have not demonstrated superiority over angiography-guided strategies in direct randomized comparisons. Intracoronary imaging offers unique information on plaque vulnerability and PCI optimization, although dedicated outcome trials in STEMI remain limited. The timing of complete revascularization has also evolved, with contemporary trials supporting early treatment in selected stable patients but not establishing a universal immediate strategy. This review summarizes current evidence, unresolved controversies, and emerging directions regarding strategies and timing of complete revascularization in STEMI patients with multivessel disease.

## 1. Introduction

Cardiovascular disease (CVD) remains the leading cause of mortality and morbidity globally, despite advances in invasive and non-invasive management [1]. Within this framework, coronary artery disease (CAD) represents the most prevalent cardiovascular (CV) clinical scenario and a major contributor to CV morbidity and mortality [2,3]. Clinically, CAD manifests as ischemic heart disease, encompassing both chronic coronary syndrome (CCS) and acute coronary syndrome (ACS) [4,5]. According to the European Society of Cardiology (ESC) guidelines, ACS describes an acute clinical event precipitated by atherosclerotic plaque destabilization, characterized by plaque rupture, fissuring, or thrombus formation, ultimately leading to myocardial infarction (MI), which is further categorized into ST-segment elevation myocardial infarction (STEMI) and non-ST-segment elevation myocardial infarction (NSTEMI). These two MI entities differ in epidemiology, pathophysiology, and, notably, treatment strategies.

Primary percutaneous coronary intervention (PCI) of the infarct-related artery remains the cornerstone of reperfusion therapy in STEMI [5,6,7].

However, the angiographic finding of multivessel CAD at the time of primary PCI is common and profoundly influences prognosis, procedural planning, and post-infarction management [8,9]. Multivessel disease is generally defined as the presence of significant stenosis in at least two major epicardial coronary arteries, usually involving vessels of at least 2.0–2.5 mm in diameter. Although definitions vary across trials, most contemporary studies have considered non-culprit lesions clinically relevant when diameter stenosis is at least 70% by visual estimation (or 50% for left main), or 50–69% when associated with functional significance [10].

**Figure 1:**
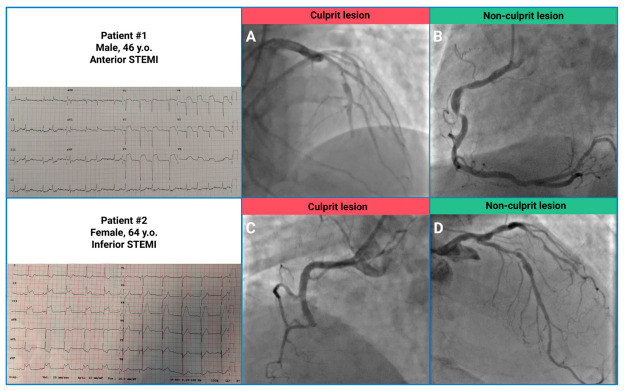
STEMI patients with multivessel disease. Patient 1. In a 46-year-old male hemodynamically stable patient with anterior STEMI, the culprit lesion was found in a left anterior descending artery thrombotic occlusion (**A**). In addition, subtotal occlusion of the large dominant right coronary artery was found (**B**). Patient 2. In a 64-year-old female patient with inferior STEMI, the culprit was complete occlusion of the right coronary artery (**C**). Furthermore, a significant calcified long lesion was detected at the mid-segment of the left anterior descending artery (**D**). STEMI: ST-elevation myocardial infarction.

It is estimated that approximately 40–50% of patients presenting with STEMI have multivessel coronary disease (Figure 1) [11,12].

The clinical relevance of multivessel disease in STEMI extends beyond the mechanical presence of additional stenoses. Patients with STEMI and multivessel disease frequently have a more diffuse and active atherosclerotic phenotype, a higher prevalence of traditional CV risk factors, a larger burden of jeopardized myocardium, and, for these reasons, are at increased risk of mortality [13]. In addition, the acute inflammatory and thrombotic milieu of STEMI may involve the entire coronary tree, supporting the concept of “pancoronary instability” [14]. Intracoronary imaging studies have demonstrated that non-culprit plaques may share features of vulnerability with the culprit lesion, including large lipid pools, thin fibrous caps, macrophage accumulation, plaque rupture, and high plaque burden. Thus, non-culprit lesions are not merely incidental angiographic findings, and in selected patients, they represent potential substrates for recurrent myocardial infarction (MI) and unplanned revascularization [15,16,17].

Historically, the interventional approach in STEMI was conservative: operators prioritized rapid restoration of flow in the infarct-related artery and avoided treatment of non-culprit vessels during the acute procedure because of concerns regarding prolonged ischemic time, greater contrast exposure, higher risk of periprocedural complications, and uncertainty about the reliability of lesion assessment during STEMI. This approach was particularly reinforced by the principle that primary PCI should be simple, rapid, and focused on the culprit artery. However, the progressive improvement in stent technology, antithrombotic therapy, radial access, intracoronary imaging, and procedural safety has allowed a more comprehensive strategy to be tested in randomized clinical trials (RCTs) [18,19,20,21].

The central question has therefore evolved from whether the culprit artery should be treated first, as an uncontested priority, to whether, how, and when non-culprit lesions should be treated after successful primary PCI. Current evidence supports complete revascularization in hemodynamically stable STEMI patients with multivessel disease, but the practical implementation of this strategy remains nuanced. Complete revascularization may be anatomical, functional, or imaging-informed. It may be performed immediately during the index primary PCI, staged during the same hospitalization, or deferred after discharge within a predefined time window [22]. It may be inappropriate in patients with cardiogenic shock (CS), very complex anatomy, severe renal dysfunction, uncertain myocardial viability, chronic total occlusions supplying limited viable myocardium, or prohibitive bleeding risk [23,24].

Therefore, complete revascularization should not be interpreted as an indiscriminate mandate to treat every angiographic abnormality. Rather, it should be understood as a structured strategy aimed at reducing residual ischemic and plaque-related risk while preserving procedural safety. In STEMI patients with multivessel disease, the decision requires integration of patient stability, lesion complexity, renal function, anticipated contrast volume, thrombotic burden, left ventricular function, ischemic territory, available expertise, and patient preference [25].

The current ESC guidelines on the management of ACS recommend complete revascularization during the index PCI procedure or within 45 days for hemodynamically stable STEMI patients with multivessel disease (Class I, Level of Evidence A), whereas angiography-guided assessment of non-culprit lesions is recommended as the preferred strategy (Class I, Level of Evidence B), without any potential benefit for functional assessment (Class III, Level of Evidence C). Conversely, in STEMI patients presenting with CS, culprit-lesion-only PCI during the index procedure remains the recommended approach (Class I, Level of Evidence B), with consideration of staged PCI of non-culprit lesions after clinical stabilization (Class IIa, Level of Evidence C) [5].

In this review, we summarize the available evidence regarding strategies and timing of complete revascularization in STEMI patients with multivessel disease. Whenever practical management considerations are discussed, we explicitly distinguish between conclusions supported by randomized clinical trials, recommendations endorsed by contemporary guidelines, and the authors’ interpretation of the available literature intended to provide a clinically oriented perspective.

## 2. Methods

We searched MEDLINE (from its inception to May 2026). We used the search terms “complete revascularization” in combination with the terms “ST-elevation myocardial infarction”, “multivessel coronary disease,” and “non-culprit lesion”. We selected publications from the past 30 years, but did not exclude commonly referenced and highly regarded older publications. We also searched the reference lists of articles identified by this search strategy and selected those we judged relevant. Review articles were cited when deemed useful to provide additional background and context. Given the narrative nature of this review, priority was assigned to RCTs, meta-analyses, and contemporary guideline documents with the greatest impact on current clinical practice. Whenever available, evidence derived from STEMI-specific populations was preferentially considered, whereas studies enrolling broader acute MI or ACS populations were included only when addressing clinically relevant questions not adequately covered by STEMI-specific data. In those cases, findings from these studies are discussed within their original clinical context and interpreted cautiously when extrapolated to STEMI-specific management.

## 3. Strategies of Complete Revascularization

### 3.1. Angiography-Guided Complete Versus Culprit-Only Revascularization

Angiography-guided complete revascularization represents the most intuitive and widely available approach to non-culprit lesion management. The strategy relies on visual or quantitative assessment of stenosis severity and generally targets non-culprit lesions with severe angiographic narrowing, most commonly ≥ 70%, or ≥50% in two projections in earlier trials. Its main advantages are simplicity, immediate applicability, and broad reproducibility in primary PCI networks. Its limitations include interobserver variability, overestimation of stenosis severity in the acute STEMI setting (by up to 10%), inability to define functional significance, and limited capacity to identify plaque vulnerability beyond lumen narrowing [26].

The first randomized signals supporting complete revascularization emerged from relatively small studies. In the trial by Politi et al., 214 patients with STEMI and multivessel disease were randomized to culprit-only PCI, staged complete revascularization, or immediate complete revascularization. Complete revascularization, whether immediate or staged, significantly reduced major adverse cardiac events (MACE) compared with culprit-only PCI (20.0% vs. 23.1% vs. 50.0%, *p* < 0.001), with the benefit largely driven by fewer repeat revascularizations and rehospitalizations. Although the study was small and conducted before several contemporary procedural refinements, it challenged the prevailing conservative paradigm [27].

Subsequently, several landmark RCTs contributed to establishing the evidence base supporting an angiography-guided approach to complete revascularization.

In the PRAMI (Randomized Trial of Preventive Angioplasty in Myocardial Infarction) trial, 465 patients with STEMI undergoing successful culprit-lesion PCI were randomized to preventive PCI of angiographically significant non-culprit lesions (>50% stenosis evaluated at visual estimation) or no preventive PCI. Preventive PCI was performed during the index procedure and targeted lesions considered severe by angiography. The trial was stopped prematurely because of a marked reduction in the primary composite endpoint of cardiac death, nonfatal MI, or refractory angina occurring in 8.9% in the intervention group compared with 22.9% in the control group (HR: 0.35, 95% confidence interval [CI]: 0.21–0.58, *p* < 0.001). Importantly, reductions were observed not only in revascularization-driven outcomes but also in recurrent MI. Nevertheless, the open-label design, early termination, inclusion of refractory angina in the endpoint, and absence of systematic physiology left uncertainty regarding the true magnitude and mechanism of benefit [28]. 

The CvLPRIT (Complete Versus Lesion-Only Primary PCI Trial) trial broadened this evidence by allowing complete revascularization either during the index procedure or before discharge. In this cohort of 296 STEMI patients, complete revascularization reduced the composite of death, recurrent MI, heart failure (HF), or ischemia-driven revascularization at one year compared with culprit-only PCI (10.0% vs. 21.2%, HR: 0.45, 95% CI: 0.24–0.84, *p* = 0.009). The trial was not powered for individual components, but it strengthened the concept that in-hospital complete revascularization could be beneficial and safe in stable STEMI patients [29].

The COMPLETE (Complete versus Culprit-Only Revascularization Strategies to Treat Multivessel Disease after Early PCI for STEMI) trial remains the pivotal study establishing complete revascularization as the default strategy in stable STEMI with multivessel disease. More than 4000 patients were randomized after successful culprit-lesion PCI to complete revascularization of suitable non-culprit lesions or culprit-only PCI. Non-culprit PCI was performed either during the index hospitalization or after discharge within 45 days, according to a prespecified strategy, and non-culprit lesions were considered significant when stenosis was >70% with visual angiographic estimation or 50–69% with a fractional flow reserve (FFR) value of ≤0.80. Complete revascularization significantly reduced the co-primary endpoint of CV death or MI (7.8% vs. 10.5%, HR: 0.74, 95% CI: 0.60–0.91, *p* = 0.004) and the broader endpoint of CV death, MI, or ischemia-driven revascularization (8.9% vs. 16.7%, HR: 0.51, 95% CI: 0.43–0.61, *p* < 0.001) at 3-year follow-up. The benefit was driven mainly by fewer MIs and fewer ischemia-driven revascularizations, without a clear reduction in all-cause mortality. Importantly, the trial confirmed that the benefit of complete revascularization was not limited to symptom relief but extended to prognostically relevant ischemic events [30]. Several points from COMPLETE are essential for clinical interpretation. First, the trial enrolled hemodynamically stable patients after successful culprit-lesion PCI, and its conclusions should not be extrapolated to CS. Second, most non-culprit lesions were selected angiographically; only a minority underwent FFR, meaning the trial is best considered an angiography-dominant strategy. Third, the benefit was particularly evident in lesions with greater angiographic severity, supporting a pragmatic threshold-based approach. Finally, complete revascularization was not performed during the primary PCI itself but rather as a planned staged strategy during the index admission or within 45 days, leaving the immediate-versus-staged question unresolved.

Finally, an individual patient data meta-analysis pooled data from six RCTs (including COMPLETE, CvLPRIT, and other trials comparing angiography-guided vs. functional-guided complete revascularization) with 8836 MI patients (of whom 87% with STEMI) and showed a significant reduction in CV death and MI with the complete revascularization strategy compared with the culprit-only treatment (9.0% vs. 11.5%, HR: 0.76, 95% CI: 0.67–0.87, *p* < 0.0001) at 36-month follow-up [31].

Taken together, angiography-guided RCTs (Table 1) demonstrate that complete revascularization reduces recurrent ischemic events compared with culprit-only PCI in stable STEMI patients. This evidence has shaped guideline recommendations and clinical practice. However, angiography alone cannot always distinguish lesions that are ischemia-producing from lesions that are visually severe but physiologically silent, nor can it identify non-obstructive vulnerable plaques. These limitations have led to the investigation of physiology-guided and imaging-guided strategies.

### 3.2. Functional-Guided Complete Revascularization

Functional assessment is attractive because it aims to treat only lesions that produce ischemia. In stable coronary artery disease, FFR-guided PCI has been shown to reduce unnecessary stenting and improve outcomes compared with angiography-guided PCI. However, in STEMI, the physiological assessment of non-culprit lesions is more complex. Acute microvascular dysfunction, vasoconstriction, neurohumoral activation, and changes in coronary flow may influence pressure-derived indices. FFR may underestimate lesion severity during the acute phase because maximal hyperemia can be blunted by microvascular dysfunction, whereas resting indices such as instantaneous wave-free ratio (iFR) may also vary between the acute and convalescent phases [32].

The DANAMI-3-PRIMULTI (complete revascularization versus treatment of the culprit lesion only in patients with ST-segment elevation myocardial infarction and multivessel disease) trial was the first randomized trial to test an FFR-guided strategy in STEMI. After successful primary PCI, 627 patients were randomized to no further invasive treatment or staged FFR-guided complete revascularization before discharge. PCI was performed for non-culprit lesions with >50% of stenosis and FFR ≤ 0.80 or very severe angiographic stenosis (>90% of stenosis). FFR-guided complete revascularization significantly reduced the composite of all-cause death, nonfatal reinfarction, or ischemia-driven revascularization (13.0% vs. 22.0%, HR: 0.56, 95% CI: 0.38–0.83, *p* = 0.004), mainly because of fewer repeat revascularizations. The trial demonstrated the feasibility of physiology-guided staged complete revascularization, but did not show a significant reduction in death or reinfarction alone [33].

The subsequent larger COMPARE-ACUTE (Fractional Flow Reserve–Guided Multivessel Angioplasty in Myocardial Infarction) trial evaluated FFR-guided complete revascularization during the index procedure or hospitalization versus culprit-only PCI in 885 STEMI multivessel patients. The strategy reduced the composite of death, MI, revascularization, or cerebrovascular events at 1 year (8.0% vs. 21.0%, HR: 0.35, 95% CI: 0.22–0.55, *p* < 0.001), again principally driven by fewer revascularizations. A key methodological strength was that FFR was also measured in the conservative arm, although results were blinded. The trial suggested that non-culprit lesion physiology can stratify risk and guide safe deferral, but the open-label nature of subsequent management and the dominance of revascularization endpoints require careful interpretation [34].

Direct randomized comparisons between FFR-guided and angiography-guided complete revascularization have not demonstrated superiority of the physiology-guided strategy. 

The FLOWER-MI (Flow Evaluation to Guide Revascularization in Multivessel ST-Elevation Myocardial Infarction) trial randomized 1163 patients with STEMI and multivessel disease to FFR-guided or angiography-guided complete revascularization. FFR guidance did not significantly reduce death, MI, or urgent revascularization at one year compared with angiography guidance (5.5% vs. 4.2%, HR: 1.32, 95% CI: 0.78–2.23, *p* = 0.31). In fact, event rates were numerically higher in the FFR-guided group, although the confidence intervals were wide and compatible with both benefit and harm. One possible explanation is that some angiographically severe but FFR-negative lesions deferred in the acute setting may later become clinically relevant, either because the acute physiological measurement underestimated true severity or because plaque vulnerability rather than ischemia drives subsequent events [35].

The FRAME-AMI (Fractional Flow Reserve vs. Angiography-Guided Strategy for Management of Non-Infarction Related Artery Stenosis in Patients with Acute Myocardial Infarction) trial offered a contrasting signal. In this trial, 562 patients with acute MI (of whom 47% with STEMI) and multivessel disease were randomized to FFR-guided versus angiography-guided PCI of non-culprit lesions. The FFR-guided strategy reduced the composite of death, MI, or repeat revascularization at longer follow-up (7.4% vs. 19.7%, HR: 0.43, 95% CI: 0.25–0.75, *p* = 0.003). However, the trial was stopped early, and enrolled a mixed acute myocardial infarction population, with STEMI representing less than half of the study cohort [36].

A neutral result of functional-guided complete revascularization was also found in the FULL-REVASC (FFR-Guidance for Complete Nonculprit Revascularization) trial. Indeed, in 1542 STEMI (88%) and very-high-risk NSTEMI patients with multivessel disease, FFR-guided complete revascularization was not shown to result in a lower risk of a composite of death from any cause, MI, or unplanned revascularization than culprit-lesion-only PCI at 4.8 years (19.0% vs. 20.4%, HR: 0.93, 95% CI: 0.74–1.17, *p* = 0.53) [37].

In the specific scenario of the elderly population, the FIRE (Functional Assessment in Elderly MI Patients with Multivessel Disease) trial enrolled 1445 older patients (≥75 years) with acute MI (35% with STEMI). In this cohort, after the culprit lesion PCI, functional-guided revascularization through FFR, non-hyperemic methods, or quantitative flow ratio (QFR) led to a significant reduction in the primary composite endpoint of death, MI, stroke, or any revascularization (15.7% vs. 21.0%, HR: 0.73, 95% CI: 0.57–0.93, *p* = 0.01) compared with no further revascularization [38]. Nevertheless, because only approximately one-third of enrolled patients presented with STEMI, the applicability of these findings to a purely STEMI population should be interpreted with appropriate caution.

More recently, trials have explored alternatives to FFR. iFR-guided complete revascularization has theoretical advantages because it avoids adenosine and may be simpler in acute settings [39]. The iMODERN (iFR-Guided Multivessel Revascularization during Percutaneous Coronary Intervention for Acute Myocardial Infarction) trial compared immediate iFR-guided PCI of non-culprit lesions with deferred cardiac stress magnetic resonance-guided revascularization in 1146 STEMI patients after successful primary PCI. Immediate iFR-guided PCI was not superior to deferred stress magnetic resonance imaging (MRI)-guided management for the composite of death, recurrent MI, or HF-related hospitalization (9.3% vs. 9.8%, HR: 0.95, 95% CI: 0.65–1.40, *p* = 0.81), suggesting that a selective deferred functional imaging strategy may be safe in selected patients [40].

Overall, functional guidance can reduce the number of treated lesions and may be particularly useful for intermediate non-culprit stenoses [25]. However, available randomized evidence (Table 2) does not consistently demonstrate superiority of physiology-guided over angiography-guided complete revascularization in STEMI patients with multivessel disease. Accordingly, current ESC guidelines favor an angiography-guided approach for non-culprit lesion treatment [5]. Nevertheless, based on the collective interpretation of available trials, physiological assessment may remain useful in selected patients with angiographically intermediate non-culprit lesions, particularly when performed during a staged procedure after partial recovery of coronary microvascular function [41]. This consideration should be regarded as an expert interpretation of the available evidence rather than a guideline-supported recommendation.

### 3.3. Intracoronary Imaging-Guided Complete Revascularization

Intracoronary imaging provides information that cannot be obtained from angiography or physiology alone [42]. Intravascular ultrasound (IVUS) allows assessment of plaque burden, vessel size, lesion length, remodeling, and stent expansion. By contrast, optical coherence tomography (OCT) offers high-resolution visualization of fibrous cap thickness, lipid arc, macrophage accumulation, thrombus, plaque rupture, erosion, calcification, dissection, and stent-related complications. Finally, near-infrared spectroscopy (NIRS) can identify lipid-rich plaques [43,44]. In STEMI, these modalities are relevant for two distinct purposes: identifying high-risk non-culprit plaques and optimizing PCI results [45].

The PROSPECT (a prospective natural-history study of coronary atherosclerosis) study, conducted in ACS patients (697, of whom 30% with STEMI), demonstrated that future adverse events frequently arise from untreated non-culprit lesions that are angiographically mild but have high-risk IVUS features, including large plaque burden (≥70%), small minimal luminal area (≤4 mm^2^), and thin-cap fibroatheroma (TCFA) phenotype. Although not restricted to STEMI, PROSPECT provided a conceptual framework: the risk of future events is determined not only by lumen severity but also by plaque biology [16].

The CLIMA (Relationship Between OCT Coronary Plaque Morphology and Clinical Outcome) study further emphasized the prognostic significance of OCT-defined plaque morphology. In this study, specific OCT features in the proximal left anterior descending artery (LAD), including minimal lumen area ≤ 3.5 mm^2^, fibrous cap thickness ≤ 75 μm, lipid arc > 180°, and macrophage infiltration, were associated with subsequent cardiac events. These findings support the hypothesis that imaging can identify plaques at high risk for future instability even when angiographic stenosis is not critical [15].

In the COMPLETE OCT substudy, STEMI patients with multivessel disease underwent OCT evaluation before PCI of non-culprit lesions. Approximately 50% of patients in this cohort had at least one obstructive non-culprit lesion with TCFA features, and vulnerable plaque characteristics were more common in angiographically obstructive lesions. This observation offers a mechanistic explanation for the clinical benefit of complete revascularization in COMPLETE: angiographically significant non-culprit lesions may not only be flow-limiting but also biologically unstable [17].

Aiming to investigate the prognostic significance of plaque vulnerability through the OCT in diabetic patients with intermediate coronary lesions, non-significant according to the FFR, the COMBINE OCT-FFR (Optical Coherence Tomography Morphologic and Fractional Flow Reserve Assessment in Diabetes Mellitus Patients) trial was designed. It enrolled 550 patients (11.2% with MI) who underwent combined functional and imaging assessment. Among FFR-negative lesions, nearly one-quarter exhibited a thin-cap fibroatheroma (TCFA) on OCT. Despite the absence of flow-limiting ischaemia, patients with TCFA showed a markedly higher incidence of major adverse cardiovascular events (MACE) at 18 months compared with those without TCFA (13.3% vs. 3.1%), with TCFA emerging as the strongest independent predictor of adverse outcomes. Overall, the trial demonstrated that plaque vulnerability may confer substantial residual risk beyond physiological lesion assessment alone, particularly in diabetic patients, thereby supporting the complementary role of intracoronary imaging for improved risk stratification [46].

The PROSPECT II (identification of vulnerable plaques and patients by intracoronary near-infrared spectroscopy and ultrasound) study, using combined NIRS and IVUS after successful PCI of flow-limiting lesions, confirmed that lipid-rich plaque and large plaque burden are associated with future coronary events [47]. More recent observational evidence integrating OCT morphology and angiography-derived physiology suggests that OCT-defined TCFA may be a stronger predictor of adverse outcomes than physiology alone in non-culprit lesions after acute myocardial infarction. This raises the possibility that a future strategy may combine ischemia assessment and plaque vulnerability assessment, rather than treating them as competing paradigms [48]. 

Despite these advances, the identification of a vulnerable non-culprit plaque does not automatically translate into a clear treatment indication. Indeed, one of the major unresolved questions in contemporary interventional cardiology is whether imaging-defined vulnerable plaques that are not flow-limiting should undergo preventive PCI or be managed with intensified medical therapy alone [49]. 

Current evidence supports the prognostic value of vulnerable plaque features identified by OCT, IVUS, and NIRS, but there is no definitive randomized evidence demonstrating that preventive PCI of non-ischemic vulnerable plaques improves clinical outcomes in STEMI patients. Importantly, plaque vulnerability and ischemic significance represent distinct biological concepts. Many future coronary events arise from lesions that are not severely obstructive, whereas many anatomically severe lesions may remain clinically silent for prolonged periods. Consequently, extrapolating risk prediction into a procedural indication remains challenging. 

At present, the most evidence-based response to the detection of vulnerable plaques is optimization of secondary prevention. Intensive lipid-lowering therapy, aggressive risk-factor control, smoking cessation, blood pressure management, glycemic optimization, and adherence to guideline-directed pharmacological therapies have all been shown to promote plaque stabilization and reduce recurrent cardiovascular events [5,14,50,51]. In this regard, intracoronary imaging may serve not only as a tool for procedural planning but also as a means of identifying patients who may derive particular benefit from intensified preventive strategies. Whether local treatment of vulnerable plaques provides incremental benefit beyond contemporary optimal medical therapy remains uncertain. Recently, the PREVENT (Preventive Coronary Intervention on Stenosis with Functionally Insignificant Vulnerable Plaque) trial has provided important insights into this debate. In 1606 patients with functionally non-significant (FFR > 0.80) but high-risk coronary plaques identified by intracoronary imaging (with either IVUS, OCT, or NIRS), preventive PCI combined with optimal medical therapy was associated with a lower incidence of MACE compared with optimal medical therapy alone (0.4% vs. 3.4%, *p* = 0.0003). Notably, the observed benefit was largely driven by reductions in unplanned revascularization, whereas differences in hard clinical endpoints such as cardiovascular death or MI were less pronounced. Although PREVENT represents a major step forward in the field of vulnerable plaque intervention, its findings should be interpreted cautiously, as questions remain regarding patient selection, lesion characteristics, procedural indications, and notably the generalizability of the results to STEMI populations, considering that only 1% of the enrolled patients in the PREVENT trial had recent STEMI (within 1 week) [52]. Consequently, whether preventive PCI should become a routine treatment strategy for imaging-defined, vulnerable, non-flow-limiting plaques remains unresolved and requires further validation in dedicated RCTs. Until additional randomized evidence becomes available, the detection of vulnerable plaques should primarily inform risk stratification, optimization of secondary prevention, and individualized clinical decision-making.

Beyond risk stratification, imaging may improve the technical quality of multivessel PCI. This is particularly important in STEMI patients, who often have heightened thrombotic activity and may require longer or more complex stent implantation when non-culprit PCI is performed. Imaging can optimize stent sizing, landing zones, calcium modification, lesion preparation, expansion, apposition, and detection of edge dissections or residual disease. These procedural advantages may be relevant when complete revascularization involves long lesions, bifurcations, calcified vessels, left main or proximal LAD disease, or diffuse atherosclerosis.

Nevertheless, imaging-guided treatment of non-culprit lesions in STEMI remains supported more by mechanistic and observational evidence than by large dedicated randomized trials (Table 3). Imaging adds time, cost, contrast use in the case of OCT, and procedural complexity. Therefore, its routine use for every non-culprit lesion cannot yet be recommended. At present, imaging appears most valuable in complex non-culprit PCI, ambiguous angiographic lesions, left main or proximal LAD disease, calcified lesions, long lesions, bifurcations, and research protocols aimed at targeting vulnerable plaques.

### 3.4. Angiography Versus Functional Versus Imaging-Guided Complete Revascularization

**Figure 2:**
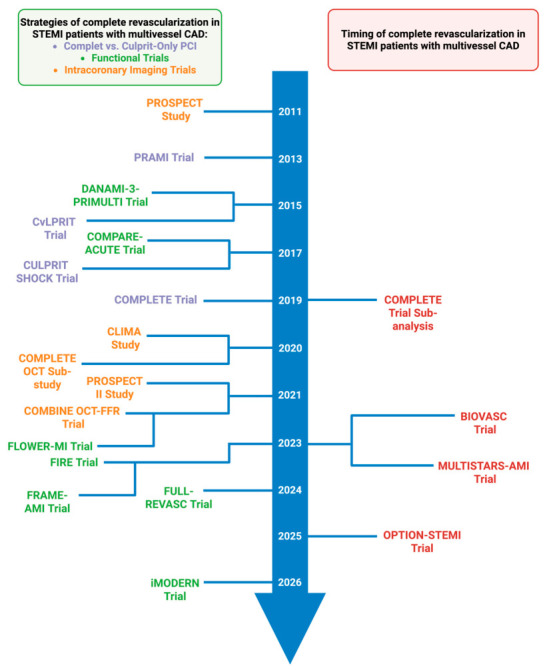
Timeline of main clinical trials addressing the strategies and timing of complete revascularization in STEMI patients with multivessel disease. CAD: coronary artery disease, PCI: percutaneous coronary intervention, STEMI: ST-elevation myocardial infarction.

Angiography, physiology, and intracoronary imaging RCTs (Figure 2) answer different questions. Angiography asks whether a lesion is visually severe. Physiology asks whether it produces ischemia. Imaging asks what the lesion is made of and whether PCI has been optimally performed. None of these approaches is universally superior because each captures only part of the pathophysiology of recurrent events after STEMI.

Angiography has the strongest randomized evidence in stable STEMI patients, largely because COMPLETE was angiography-dominant and powered for clinically meaningful outcomes. It is simple, available, fast, and well-suited to clearly severe lesions. Its weakness is that visual estimation may overestimate or underestimate true risk, particularly in intermediate lesions and diffuse disease [30].

Physiology offers a rational method for avoiding unnecessary PCI, reducing stent number, procedure length, and long-term metal burden. It is particularly useful for intermediate lesions. However, acute STEMI physiology may be unstable, and direct comparisons have not consistently shown superiority over angiography. Moreover, plaque-related events may arise from lesions that are not ischemia-producing at the time of assessment [35,36].

Imaging provides the most detailed anatomical and biological information. It can identify vulnerable plaques and optimize PCI, but it does not directly prove ischemia and lacks definitive large-scale randomized evidence for routine non-culprit lesion selection in STEMI. Its greatest value may be complementary rather than competitive: imaging can refine PCI when revascularization has already been selected and may identify high-risk plaques for future preventive strategies [15,16].

**Figure 3:**
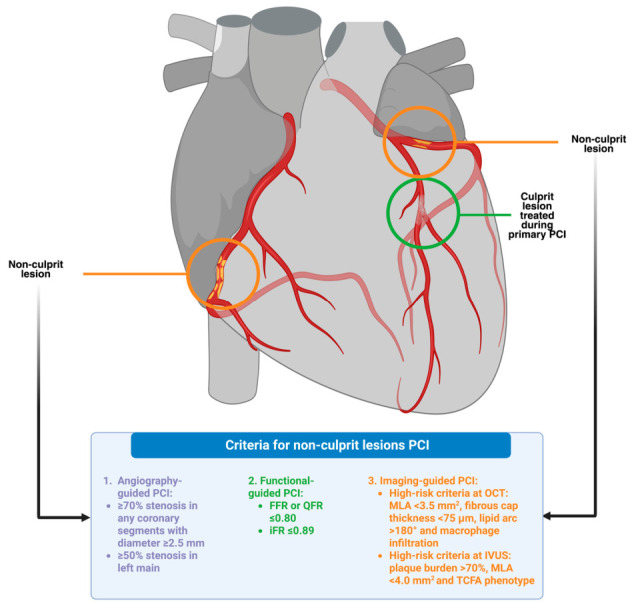
Angiography, functional and intracoronary imaging guidance criteria for performing non-culprit-lesion PCI aiming to achieve a complete revascularization in STEMI patients with multivessel disease. FFR: fractional flow reserve, iFR: instantaneous wave-free ratio, IVUS: intravascular ultrasound, MLA: minimal luminal area, OCT: optical coherence tomography, PCI: percutaneous coronary intervention, QFR: quantitative flow ratio, TCFA: thin-cap fibroatheroma.

Taken together, currently available evidence suggests that angiography, physiology, and intracoronary imaging should be viewed as complementary rather than competing modalities, and the choice of guiding strategy is therefore lesion-specific with validated criteria to proceed to the revascularization of non-culprit lesions (Figure 3). Severe non-culprit lesions in large vessels, especially proximal segments supplying substantial myocardium, can be treated on angiographic grounds. Intermediate lesions may be assessed with physiology, preferably in a staged setting. Complex lesions selected for PCI should be optimized with intracoronary imaging. Future trials will determine whether integrated strategies combining angiographic severity, functional significance, and plaque vulnerability can outperform any single modality. This conceptual framework reflects the authors’ interpretation of the available randomized evidence, observational studies, and contemporary guideline recommendations. It is intended to facilitate discussion of potential clinical scenarios and should not be interpreted as a formal recommendation or decision-making algorithm endorsed by scientific societies.

Finally, a promising strategy is the hybrid coronary revascularization, combining CABG with an internal mammary artery graft to the LAD and PCI to other non-LAD segments for the treatment of STEMI patients with multivessel disease. This strategy is supported in patients with LAD suitable for CABG and non-LAD lesions suitable for PCI with the following clinical characteristics: intermediate-high anatomical complexity (SYNTAX [SYNergy between PCI with TAXUS and Cardiac Surgery] score ≥ 23), unprotected left main lesions unsuitable for PCI, left ventricular dysfunction (LVEF ≤ 35%), diabetes mellitus, and renal failure [5,53].

## 4. Timing of Complete Revascularization: Immediate Versus Staged

The timing of complete revascularization remains one of the most debated aspects of STEMI care (Table 4). Potential advantages of immediate complete revascularization include avoidance of recurrent ischemia before staged PCI, shorter hospitalization, fewer readmissions, single arterial access, lower overall costs, and greater patient convenience. Potential disadvantages include longer primary PCI procedures, higher contrast and radiation exposure, treatment decisions during a period of vasoconstriction and microvascular dysfunction, greater thrombotic burden, operator fatigue during off-hours procedures, and increased risk in patients with renal dysfunction or complex anatomy [54].

The COMPLETE trial established that staged complete revascularization within 45 days is beneficial compared with culprit-only PCI, but it did not directly compare immediate and staged strategies. In COMPLETE, benefit was consistent whether non-culprit PCI was planned during the index hospitalization or after discharge, suggesting that complete revascularization does not necessarily need to be performed during the primary PCI procedure [55].

The BIOVASC (Percutaneous Complete Revascularisation Strategies using Sirolimus-Eluting Biodegradable Polymer-Coated Stents in Patients Presenting with Acute Coronary Syndrome and Multivessel Disease) trial was the first major randomized trial specifically addressing the timing of revascularization in ACS patients with multivessel disease. It enrolled 1525 patients (of whom 40% with STEMI) and compared immediate complete revascularization with staged complete revascularization during hospitalization or within six weeks. Immediate complete revascularization was non-inferior to staged treatment for the primary composite endpoint of all-cause mortality, MI, any unplanned ischaemia-driven revascularisation, or cerebrovascular events (7.6% vs. 9.4%, HR: 0.78, 95% CI: 0.55–1.11, *p* = 0.0011 for non-inferiority) and was associated with fewer MI (1.9% vs. 4.5%, HR: 0.41, 95% CI: 0.22–0.76, *p* = 0.0045) and unplanned ischemia-driven revascularizations (4.2% vs. 6.7%, HR: 0.61, 95% CI: 0.39–0.95, *p* = 0.030), particularly early after the index procedure. Although BIOVASC included both STEMI and NSTEMI presentations, with STEMI accounting for only 40% of the study population, the findings support the feasibility and safety of immediate treatment in selected stable patients. Nevertheless, direct extrapolation to an exclusively STEMI population should be made cautiously [56].

The MULTISTARS-AMI (Multivessel Immediate versus Staged Revascularization in Acute Myocardial Infarction) trial focused specifically on hemodynamically stable STEMI patients and enrolled 840 subjects. Immediate multivessel PCI was non-inferior and statistically superior to staged PCI performed 19–45 days later for a composite endpoint including death, MI, stroke, unplanned ischemia-driven revascularization, or HF hospitalization (8.5% vs. 16.2%, risk ratio [RR]: 0.52, 95% CI: 0.38–0.72, *p* < 0.001 for non-inferiority and *p* < 0.001 for superiority). The benefit was driven mainly by reductions in nonfatal MI and unplanned revascularization. However, the trial excluded patients with complex anatomy and high-risk clinical features, meaning that its results should be applied selectively [57].

Finally, the OPTION-STEMI (Optimal Timing of Fractional Flow Reserve-Guided Complete Revascularization for Non-Infarct-Related Artery in ST-segment Elevation Myocardial Infarction with Multivessel Disease) trial added a more cautious perspective. This large STEMI-centered trial enrolled 994 patients and showed that immediate complete revascularization was not shown to be non-inferior to staged complete revascularization during the index hospitalization for the composite of all-cause death, nonfatal MI, or unplanned revascularization at 1-year (13% vs. 11%, HR: 1.24, 95% CI: 0.86–1.79, *p* for non-inferiority = 0.24) [58]. However, it should be noted that in the enrolled cohort > 30% of included patients had acute decompensated HF and, in this respect, treatment effect varied according to Killip class, with an increased risk of harm from immediate complete revascularisation for patients with a higher Killip class (II or III) than for those without clinical signs of HF (Killip I; *p* for interaction = 0.04) in a consistent way with the findings of the CULPRIT-SHOCK trial [23]. The findings suggest that although immediate complete revascularization is feasible and may be appropriate in low-risk, stable patients with simple lesions, staged complete revascularization remains a robust and prudent strategy, particularly when clinical or anatomical uncertainty exists [58].

Finally, a recent meta-analysis pooled data from 9 RCTs (including BIOVASC, MULTISTARS-AMI, and OPTION-STEMI trials) with 4213 STEMI patients and multivessel disease. The analysis revealed that in hemodynamically stable STEMI patients, immediate complete revascularization was associated with an increased short-term cardiac death (RR: 2.19, 95% CI: 1.08–4.44, *p* = 0.03) compared with staged complete revascularization. A nonsignificant trend toward an increased short-term (2.0% vs. 1.2%, RR: 1.66, 95% CI: 0.99–2.78, *p* = 0.053) and long-term (4.7% vs. 3.5%, incident rate ratio: 1.40, 95% CI: 0.97–2.03, *p* = 0.07) all-cause mortality was also found, while no differences were found in long-term (1-year) mortality neither in short- or long-term follow-up for MI, repeat revascularization, stent thrombosis, stroke, major bleeding and the composite of MACEs [59]. The mechanisms underlying this finding require careful consideration. Immediate complete revascularization inevitably prolongs the duration of the index procedure and increases contrast administration, radiation exposure, and procedural complexity. Although these factors may have limited consequences in low-risk patients, they could become clinically relevant in subjects with impaired left ventricular function, early heart failure, borderline hemodynamic status, or renal dysfunction. Furthermore, non-culprit lesion assessment performed during the acute phase of STEMI may be affected by transient vasoconstriction, systemic inflammatory activation, endothelial dysfunction, and microvascular impairment [41]. As a consequence, some lesions selected for immediate treatment may not represent the optimal targets for intervention during the index procedure. Additional PCI in non-culprit vessels may also increase the risk of distal embolization, peri-procedural myocardial injury, side-branch compromise, and transient hemodynamic deterioration during a period in which myocardial reserve is already substantially reduced [54]. The findings of OPTION-STEMI further support this hypothesis [58]. Although these observations should be interpreted cautiously, they suggest that the physiological tolerance to prolonged and more extensive procedures may differ substantially across STEMI phenotypes. From a clinical standpoint, these data argue against considering immediate complete revascularization as a universal strategy. Rather, they support a more selective approach in which immediate treatment may be appropriate for hemodynamically stable patients with simple non-culprit anatomy, low anticipated procedural burden, preserved renal function, and absence of clinical HF. Conversely, when procedural complexity, contrast volume, or clinical instability are concerns, staged revascularization may provide a safer strategy while preserving the long-term benefits associated with complete revascularization. Taken together, available randomized trials provide heterogeneous results regarding the optimal timing of complete revascularization. While studies such as BIOVASC and MULTISTARS-AMI support the feasibility of immediate complete revascularization in selected stable patients, OPTION-STEMI and recent meta-analytic evidence suggest that a staged approach remains a valid and potentially safer strategy in higher-risk clinical settings. Therefore, current evidence supports an individualized decision-making process. The clinical considerations discussed below represent the authors’ interpretation of the available literature and should be integrated with contemporary guideline recommendations and patient-specific factors. Immediate complete revascularization may be considered when the culprit PCI is uncomplicated, the patient is stable, renal function is preserved, contrast use is modest, non-culprit anatomy is simple, stenoses are clearly severe, and operator and cath lab conditions are optimal. Staged PCI is preferable when culprit PCI is prolonged or complicated, contrast volume is high, renal function is impaired, lesions are complex, thrombus burden is substantial, physiology or imaging is required, or there is uncertainty regarding the clinical relevance of non-culprit disease [54]. When staged, revascularization should generally be completed during the index hospitalization or within 45 days, consistent with the ESC guidelines recommendations [5,55].

## 5. Complete Revascularization in Cardiogenic Shock

CS affects up to 10% of patients with MI, occurring more frequently in the setting of complete coronary occlusion, and continues to be associated with high mortality and morbidity [60,61]. In this framework, an immediate revascularization strategy, with either PCI or coronary artery bypass graft (CABG), was associated with a significant reduction in 6-month mortality in the SHOCK (SHould we emergently revascularize Occluded Coronaries for cardiogenic shocK?) trial [62,63]. For this reason, immediate PCI of the culprit artery is recommended in STEMI-related CS. Alternatively, if PCI is not feasible or unsuccessful, emergency CABG is likewise recommended [5]. However, CS represents a distinct clinical scenario in which the evidence supporting complete revascularization in stable STEMI does not apply. In shock, multivessel disease is common and identifies a particularly high-risk phenotype.

The theoretical rationale for immediate multivessel PCI is attractive: restoring flow in multiple territories may improve global myocardial perfusion and hemodynamics. However, prolonged procedures, higher contrast volume, greater ischemic time, thrombotic complications, distal embolization, arrhythmias, and renal injury may all worsen shock physiology.

The CULPRIT-SHOCK (Culprit Lesion-Only PCI versus Multivessel PCI in Cardiogenic Shock) trial fundamentally changed practice. A total of 706 patients with acute MI, multivessel disease, and CS were randomized to culprit-lesion-only PCI with possible staged revascularization or immediate multivessel PCI. Culprit-lesion-only PCI reduced the composite of death or severe renal failure requiring renal replacement therapy at 30 days, mainly through lower mortality. At one year, repeat revascularization and HF hospitalization were more frequent in the culprit-only group, but the early survival signal established culprit-only PCI as the preferred initial approach [23].

Several mechanisms may explain these findings. In CS, procedural simplicity and rapid restoration of culprit-vessel flow are paramount. Treating additional lesions can increase contrast load and worsen renal function, prolong procedural time during severe hemodynamic instability, expose non-culprit territories to periprocedural complications, and aggravate systemic inflammation, vasoplegia, and multiorgan failure. Therefore, routine immediate complete revascularization should be avoided in CS.

Furthermore, it should be noted that only two-thirds of the patients in the CULPRIT-SHOCK trial had STEMI, and only 12% received a left ventricular unloading with a microaxial flow pump Impella, which recently emerged as a beneficial device in STEMI-related CS in the DanGer-SHOCK (Danish-German Cardiogenic Shock) trial [64]. In this respect, a pre-specified sub-analysis of the trial revealed that, in the DanGer-SHOCK cohort, immediate complete multivessel PCI was associated with a significant reduction in 180-day all-cause mortality compared with culprit-only PCI (50% vs. 61%, adjusted odds ratio [OR]: 0.40, 95% CI: 0.19–0.83) [65]. These findings could unravel the potential for the immediate complete revascularization strategy also in the CS clinical scenario, if performed during left ventricular unloading with the Impella device. Importantly, these observations should be considered hypothesis-generating, as they derive from a selected CS population and should be regarded as exploratory when applied to the broader STEMI population. At present, the recommendation for culprit-lesion-only PCI in patients with MI complicated by CS remains primarily grounded in randomized evidence from CULPRIT-SHOCK and is reflected in current guideline recommendations [5]. Any extension of complete revascularization strategies to mechanically supported CS populations requires confirmation in dedicated randomized trials.

This does not mean that non-culprit lesions should never be treated in shock. Selective intervention may be justified when the non-culprit lesion is flow-limiting in a vessel supplying a large viable territory, when persistent ischemia remains after culprit PCI, when the culprit vessel is uncertain, or when revascularization of another critical lesion is expected to stabilize hemodynamics. 

Otherwise, staged evaluation after recovery is preferred, ideally with Heart Team input for complex anatomy, left main disease, chronic total occlusions, or consideration of surgical revascularization.

## 6. Implications on Antithrombotic Therapy

Antithrombotic therapy is an integral component of the complete revascularization strategy in STEMI patients with multivessel disease, as the extent and complexity of coronary intervention may directly influence the balance between ischemic protection and bleeding risk [66]. Complete revascularization in STEMI with multivessel disease has important implications for antithrombotic therapy and, notably, for dual antiplatelet therapy (DAPT). These patients are at high ischemic risk because they combine an acute thrombotic presentation, diffuse atherosclerosis, multiple treated or untreated plaques, and often more complex PCI [67]. At the same time, more extensive PCI may increase bleeding risk indirectly by reinforcing the perceived need for prolonged or intensified antithrombotic therapy [20]. Indeed, ESC guidelines consider multivessel CAD as a high-risk clinical feature to extend DAPT or dual antithrombotic therapy over 12 months in patients without high bleeding risk features (Class IIa, Level of Evidence A) [5].

In the acute phase, potent P2Y12 inhibition with ticagrelor or prasugrel in addition to aspirin remains the default strategy for STEMI patients undergoing primary PCI, unless contraindications or high bleeding risk favor clopidogrel. Adequate periprocedural anticoagulation is essential, and glycoprotein IIb/IIIa inhibitors may be reserved for bailout situations such as large thrombus burden, no-reflow, or thrombotic complications [5]. Because complete revascularization frequently requires treatment of multiple lesions and longer procedural duration, achieving rapid and consistent platelet inhibition may be particularly relevant during the peri-procedural phase [68,69]. In this context, intravenous cangrelor may represent a useful option in selected high-risk patients, especially when immediate multivessel PCI is performed or when oral P2Y12 inhibitor absorption is uncertain, even more so in the CS scenario [70]. When non-culprit PCI is staged within days or weeks, antithrombotic continuity becomes central. Patients should remain protected by DAPT between procedures, especially because untreated severe non-culprit lesions may remain substrates for recurrent events. Conversely, if staged PCI is delayed, clinicians must ensure adherence and avoid premature de-escalation in patients with high ischemic burden. Complete revascularization often overlaps with procedural features traditionally associated with complex PCI, including multiple treated vessels, greater stent length, bifurcation treatment, and multilesion intervention [71]. Consequently, antithrombotic management should be individualized according to both patient-related and procedure-related ischemic and bleeding risks [20].

In this respect, the DAPT-MVD (Dual Antiplatelet Therapy in Patients With Coronary Multi-Vessel Disease) trial enrolled 8250 patients with multivessel CAD who completed 12 months of DAPT, randomizing them to receive DAPT for an additional year or to continue with only aspirin. The primary endpoint of MACE was slightly reduced by the extended DAPT (5.8% vs. 6.8%, HR: 0.82; 95% CI: 0.69–0.98, *p* = 0.03) without any differences in terms of major bleeding, showing that prolonged platelet inhibition offers modest but meaningful protection when treatment tolerance and a low bleeding risk are present [72].

In a similar way, the PARTHENOPE (Personalized or Standard Duration of Dual Antiplatelet Therapy After Percutaneous Coronary Intervention) trial compared personalized versus standard DAPT duration after PCI, reinforcing the concept that a uniform antiplatelet strategy may be suboptimal after contemporary PCI. In this cohort, the personalized DAPT was superior to standard DAPT in patients with high ischemic risk (revealed with DAPT score > 2), with the greatest treatment effect observed in patients with STEMI, diabetes, and higher DAPT scores, which may also serve as a proxy for the frequent coexistence of multivessel disease [73].

Patients requiring oral anticoagulation represent an additional challenge. In STEMI patients with atrial fibrillation or another indication for anticoagulation, the need for multivessel PCI increases ischemic concern but does not eliminate the bleeding hazard of triple therapy. Contemporary practice generally favors the shortest feasible duration of triple therapy followed by oral anticoagulation plus a P2Y12 inhibitor, usually clopidogrel, with treatment individualized according to stent thrombosis risk, bleeding risk, and procedural complexity [5,74].

Therefore, antithrombotic therapy should be considered an integral component of the complete revascularization strategy rather than a separate treatment domain. Decisions regarding DAPT intensity and duration should be guided not only by the acute STEMI presentation but also by the extent of revascularization achieved, procedural complexity, residual ischemic risk, and individual bleeding susceptibility.

## 7. Ongoing Evidence

Interpretation of the available evidence requires recognition of the substantial heterogeneity across contemporary trials. Studies differed considerably with respect to lesion-selection criteria, timing definitions, proportions of STEMI patients, procedural complexity, and endpoint composition. Importantly, several positive trials derived much of their benefit from reductions in repeat revascularization rather than from reductions in mortality or recurrent MI. Moreover, many studies were not powered to detect differences in hard clinical endpoints, which may partly explain the apparently divergent results observed across trials evaluating immediate and staged complete revascularization. Several ongoing or recently completed trials are expected to refine the management of non-culprit lesions in STEMI. The INTERCLIMA (NCT05027984) trial and related imaging-based studies are expected to clarify whether OCT-defined or multimodality-defined vulnerable plaques should be treated pre-emptively, particularly when they are not physiologically significant. The COMBINE-INTERVENE (NCT05333068) trial builds on the concept that vulnerable plaque morphology and functional significance may provide complementary risk stratification, potentially identifying a subgroup in whom PCI of non-flow-limiting but high-risk plaques could improve outcomes.

**Figure 4:**
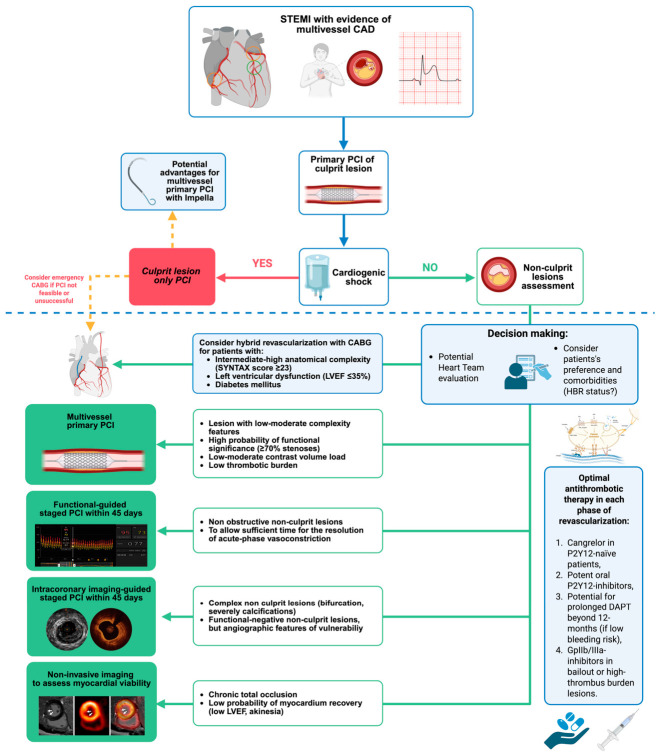
Treatment algorithm for complete revascularization in STEMI patients with multivessel disease. CABG: coronary artery bypass grafting, CAD: coronary artery disease, DAPT: dual antiplatelet therapy, HBR: high bleeding risk, LVEF: left ventricular ejection fraction, PCI: percutaneous coronary intervention, SYNTAX: SYNergy between PCI with TAXUS and cardiac surgery, STEMI: ST-elevation myocardial infarction.

Other trials will address guidance and timing more directly. The COMPLETE-2 (NCT05701358) trial is evaluating physiology-guided versus angiography-guided non-culprit lesion PCI and includes an OCT observational component, potentially helping to reconcile ischemia-based and plaque-based models. AIR-STEMI (NCT05818475) is testing angiography-derived FFR guidance against angiographic guidance in STEMI, while FAVOR-V (NCT05669222) is evaluating angiography-derived physiology and biomechanical plaque metrics [75]. OCT-CONTACT (OCT-guided vs. COmplete PCI in patieNts with sT-segment elevation myocArdial infarCtion and mulTivessel disease) and VULNERABLE (NCT05599061) are exploring imaging-guided approaches to identify and treat high-risk plaques [76]. Finally, the STAGED (staged interventional strategies for acute ST-segment elevation myocardial infarction patients with multivessel disease) and TERMINAL (NCT05231226) trials are investigating different staged and immediate timing strategies [77]. Collectively, these trials may shift the field from the binary question of complete versus culprit-only revascularization toward a precision model in which lesion selection, PCI optimization, timing, and antithrombotic intensity are integrated. To summarize the available evidence, we propose a conceptual therapeutic framework integrating findings from randomized trials, contemporary guideline recommendations, and areas in which clinical judgment remains necessary because of limited evidence. This figure reflects the authors’ interpretation of the current literature, and it should not be considered a formal guideline-endorsed treatment algorithm (Figure 4).

## 8. Conclusions

In hemodynamically stable STEMI patients with multivessel disease, complete revascularization has become the preferred strategy after successful primary PCI of the culprit artery. The strongest evidence supports treatment of angiographically significant non-culprit lesions, with reductions in MI and ischemia-driven revascularization compared with culprit-only PCI. Physiology-guided complete revascularization can reduce unnecessary PCI and is useful for intermediate lesions, but direct comparisons with angiography-guided strategies have produced inconsistent results. Intracoronary imaging offers a biologically compelling approach by identifying vulnerable plaques and optimizing PCI, although definitive randomized outcome evidence in STEMI remains incomplete.

The timing of complete revascularization should be individualized. Immediate complete revascularization is reasonable in selected stable patients with simple anatomy, preserved renal function, and clearly severe non-culprit lesions. Staged complete revascularization remains appropriate, particularly when lesions are complex, procedural risk is high, or additional functional or imaging assessment is needed. In CS, routine immediate multivessel PCI should be avoided; culprit-lesion-only PCI with selective staged revascularization remains the evidence-based approach.

The future of STEMI multivessel disease management will likely focus on refining patient and lesion selection while preserving angiography-guided complete revascularization as the evidence-based standard approach. Additional physiological and intracoronary imaging assessment may provide incremental value in selected clinical scenarios, particularly when lesion significance or plaque characteristics remain uncertain. Complete revascularization should therefore be pursued as an evidence-based strategy aimed at reducing recurrent ischemic risk while balancing procedural safety and long-term clinical outcomes.

## Figures and Tables

**Table 1:** Main randomized trials comparing complete revascularization versus culprit-only revascularization in STEMI patients with multivessel disease.

Trial, Year	Patients (*n*) and STEMI %	Intervention Group	Control Group	Evaluation of Non-Culprit Lesions	Primary Endpoint	Main Results
Politi et al., 2010 [27]	214 (100%)	Immediate (*n* = 65) or staged (*n* = 65) complete revascularization	Culprit-only PCI (*n* = 84)	Angiography, >70%	MACE: all-cause death, re-hospitalization for ACS, recurrent MI, repeat coronary revascularization at 30-month follow-up	20.0% staged complete vs. 23.1% immediate complete vs. 50.0% culprit-only (*p* < 0.001)
PRAMI trial, 2013 [28]	465 (100%)	Preventive PCI during index procedure (*n* = 234)	Culprit-only PCI (*n* = 231)	Angiography, ≥50% in non-culprit artery	Composite of cardiac death, nonfatal MI, or refractory angina at 23-month follow-up	8.9% vs. 22.9%; HR: 0.35 (95% CI: 0.21–0.58, *p* < 0.001)
CvLPRIT trial, 2015 [29]	296 (100%)	Complete revascularization during index procedure or admission (*n* = 150)	Culprit-only PCI (*n* = 146)	Angiography, >70% in one view or >50% in two views	All-cause death, recurrent MI, HF, ischemia-driven revascularization at 12-month follow-up	10.0% vs. 21.2%; HR: 0.45 (95% CI: 0.24–0.84, *p* = 0.009)
CULPRIT SHOCK trial, 2017 [23]	706 (62%)	Culprit-only PCI in patients with MI-related CS (*n* = 344)	Immediate multivessel PCI in patients with MI-related CS (*n* = 342)	Angiography, >70%	Composite of death or renal failure leading to renal replacement therapy at 30-day follow-up	45.9% vs. 55.4% (*p* = 0.01)
COMPLETE trial, 2019 [30]	4041 (100%)	Staged complete revascularization during admission or within 45 days (*n* = 2016))	Culprit-only PCI (*n* = 2025)	Angiography, ≥70% or angiography 50–69% and FFR ≤ 0.80	(1) CV death or MI; (2) CV death, MI, or ischemia-driven revascularization at 3-year follow-up	(1) 7.8% vs. 10.5%; HR: 0.74 (95% CI: 0.60–0.91, *p* = 0.004) (2) 8.9% vs. 16.7%; HR: 0.51 (95% CI: 0.43–0.61, *p* < 0.001)

ACS: acute coronary syndrome, CI: confidence interval, CS: cardiogenic shock, CV: cardiovascular, FFR: fractional flow reserve, HF: heart failure, HR: hazard ratio, MACE: major adverse cardiovascular events, MI: myocardial infarction.

**Table 2:** Main randomized trials comparing angiography-guided versus functional-guided complete revascularization in STEMI patients with multivessel disease.

Trial, Year	Patients (*n*) and STEMI %	Intervention Group	Control Group	Evaluation of Non-Culprit Lesions	Primary Endpoint	Main Results
DANAMI-3-PRIMULTI Trial, 2015 [33]	627 (100%)	Staged FFR-guided complete revascularization (*n* = 314)	Culprit-only PCI (*n* = 313)	Angiography, >90% or >50% with FFR ≤ 0.80	All-cause death, nonfatal re-MI, ischemia-driven revascularization at 27-month follow-up	13.0% vs. 22.0%; HR: 0.56 (95% CI: 0.38–0.83, *p* = 0.004)
COMPARE-ACUTE Trial, 2017 [34]	885 (100%)	FFR-guided complete revascularization during index procedure/admission (*n* = 295)	Culprit-only PCI (*n* = 590)	Angiography, ≥50% with FFR ≤ 0.80	Death, MI, revascularization, cerebrovascular events at 1-year follow-up	8.0% vs. 21.0%; HR: 0.35 (95% CI: 0.22–0.55, *p* < 0.001)
FLOWER-MI Trial, 2021 [35]	1163 (100%)	FFR-guided complete revascularization (*n* = 586)	Angiography-guided complete revascularization (*n* = 577)	≥50% and FFR ≤ 0.80 in FFR arm; ≥50% by angiography in control arm	Death, nonfatal MI, urgent revascularization at 1-year follow-up	5.5% vs. 4.2%; HR: 1.32 (95% CI: 0.78–2.23, *p* = 0.31)
FRAME-AMI Trial, 2023 [36]	562 (47%)	FFR-guided complete revascularization through PCI (*n* = 284)	Angiography-guided complete revascularization through PCI (*n* = 278)	≥50% and FFR ≤ 0.80 in FFR arm; ≥50% by angiography in control arm	Death, MI, repeat revascularization at 3.5-year follow-up	7.4% vs. 19.7%; HR: 0.43 (95% CI: 0.25–0.75, *p* = 0.003)
FIRE Trial, 2023 [38]	1445 (35%)	Physiology-guided complete revascularization (*n* = 720)	Culprit-only or deferred selective revascularization (*n* = 725)	Functional, FFR ≤ 0.80 or QFR ≤ 0.80 or iFR ≤ 0.89	Death, MI, stroke, or ischemia-driven revascularization at 1-year follow-up	15.7% vs. 21.0%; HR: 0.73 (95% CI: 0.57–0.93, *p* = 0.01)
FULL-REVASC Trial, 2024 [37]	1542 (88%)	FFR-guided complete revascularization (*n* = 764)	Usual care after culprit-lesion PCI (*n* = 778)	Functional, FFR ≤ 0.80	Death, MI, or unplanned revascularization at 4.8-year follow-up	19.0% vs. 20.4%; HR: 0.93 (95% CI: 0.74–1.17, *p* = 0.53)
iMODERN Trial, 2026 [40]	1146 (100%)	Immediate iFR-Guided Complete Revascularization (*n* = 558)	Deferred Stress MRI-Guided PCI (*n* = 588)	>50% with iFR ≤ 0.89 vs. Ischemia on MRI	All-cause death, recurrent MI, HF hospitalization	9.3% vs. 9.8%; HR: 0.95 (95% CI: 0.65–1.40, *p* = 0.81)

ACS: acute coronary syndrome, CI: confidence interval, CS: cardiogenic shock, CV: cardiovascular, FFR: fractional flow reserve, HF: heart failure, HR: hazard ratio, iFR: instantaneous wave-free ratio, MACE: major adverse cardiovascular events, MI: myocardial infarction, MRI: magnetic resonance imaging, QFR: quantitative flow ratio.

**Table 3:** Main studies assessing intracoronary imaging for non-culprit lesions in STEMI patients with multivessel disease.

Trial, Year	Patients (*n*) and STEMI %	Imaging Strategy	Reference Strategy	Evaluation of Non-Culprit Lesions	Primary Endpoint	Main Results
PROSPECT Trial, 2011 [16]	697 (30%)	Three-vessel grayscale and radiofrequency IVUS after successful PCI of culprit lesions	Angiographic assessment and clinical follow-up of untreated plaques	IVUS, non-culprit lesions characterized by plaque burden, MLA, and virtual-histology TCFA	MACE: cardiac death, cardiac arrest, MI, or rehospitalization for unstable/progressive angina at 3.4-year follow-up	Events were similarly attributable to culprit and non-culprit lesions. CV events frequently arose from angiographically mild lesions with high-risk IVUS features
CLIMA Study, 2020 [15]	1003 (20%)	OCT assessment of untreated proximal LAD segments	Absence of OCT-defined high-risk plaque phenotype	OCT high-risk plaque defined by MLA < 3.5 mm^2^, fibrous cap thickness < 75 μm, lipid arc > 180°, and macrophage infiltration	Cardiac death or target-segment MI at 12 months follow-up	The simultaneous presence of more OCT high-risk features identified lesions at a higher risk of CV events
COMPLETE OCT substudy, 2020 [17]	93 (100%)	OCT imaging of at least two coronary arteries before planned non-culprit lesion PCI	Angiographic stenosis severity and OCT plaque phenotype	OCT assessment of obstructive and non-obstructive non-culprit plaques, including TCFA	Prevalence and morphology of vulnerable non-culprit plaques	Nearly 50% of patients had at least one non-culprit lesion with TCFA; vulnerable plaque features were more frequent in obstructive lesions
COMBINE OCT-FFR Trial, 2021 [46]	550 (unknown, 11.2% MI)	OCT evaluation of FFR-negative non-culprit lesions	FFR-negative lesions without OCT-defined TCFA	FFR plus OCT plaque morphology in angiographically intermediate lesions	Target lesion-related MACE	OCT-defined TCFA in FFR-negative lesions identified a higher event risk
PROSPECT II Trial, 2021 [47]	898 (22%)	Three-vessel NIRS-IVUS after PCI of flow-limiting lesions	Lesions without high lipid burden or large plaque burden	NIRS lipid-core burden index and IVUS plaque burden in untreated non-culprit lesions	Non-culprit lesion-related MACE	Lipid-rich plaques with a large plaque burden predicted future non-culprit lesion-related events
OCT/μQFR AMI study, 2025 [48]	645 (70%)	Three-vessel OCT plus angiography-derived μQFR assessment of intermediate non-culprit lesions	μQFR-defined functional significance and OCT-defined plaque vulnerability	OCT-defined TCFA versus μQFR-defined physiological significance	Long-term clinical outcomes related to non-culprit lesions	OCT-defined TCFA emerged as the dominant predictor of adverse outcomes, whereas μQFR-defined functional significance was less prognostically informative

ACS: acute coronary syndrome, CV: cardiovascular, FFR: fractional flow reserve, IVUS: intravascular ultrasound, LAD: left anterior descending artery, MACE: major adverse cardiovascular events, MI: myocardial infarction, MLA: minimal luminal area, NIRS: near-infrared spectroscopy, OCT: optical coherence tomography, TCFA: thin-cap fibroatheroma.

**Table 4:** Main randomized trials comparing immediate versus staged complete revascularization in STEMI patients with multivessel disease.

Trial, year	Patients (*n*) and STEMI %	Intervention Group	Control Group	Evaluation of Non-Culprit Lesion	Primary Endpoint	Main Results
Politi et al. 2010 [27]	214 (100%)	Immediate complete revascularization (*n* = 65)	Staged complete revascularization and culprit-only arms (*n* = 65)	Angiography, >70%	MACE: all-cause death, re-hospitalization for ACS, recurrent MI, repeat coronary revascularization at 30-month follow-up	20.0% staged complete vs. 23.1% immediate complete (*p* = 0.815)
COMPLETE Timing Sub-analysis, 2019 [55]	4041 (100%)	Staged PCI during index admission	Staged PCI after discharge within 45 days	Angiography, ≥70% or 50–69% with FFR ≤ 0.80	Co-primary endpoints of CV death or MI at 4-year follow-up	Benefit versus culprit-only PCI is consistent for in-hospital and post-discharge (within 45 days) staged strategies
BIOVASC Trial, 2023 [56]	1525 (40%)	Immediate complete revascularization (*n* = 764)	Staged complete revascularization during admission or within 6 weeks (*n* = 598)	≥70% or positive physiology (FFR or iFR)	All-cause death, nonfatal MI, unplanned ischemia-driven revascularization, or cerebrovascular events at 1-year follow-up	7.6% vs. 9.4%; HR: 0.78 (95% CI: 0.55–1.11, *p* = 0.0011 for non-inferiority); fewer MI and revascularizations with the immediate complete strategy
MULTISTARS-AMI Trial, 2023 [57]	840 (100%)	Immediate multivessel PCI (*n* = 418)	Staged PCI 19–45 days later (*n* = 422)	Angiography, ≥70%	Death, nonfatal MI, stroke, unplanned ischemia-driven revascularization, HF hospitalization at 1-year follow-up	8.5% vs. 16.3%; RR: 0.52 (95% CI: 0.38–0.72, *p* < 0.001 for non-inferiority and *p* < 0.001 for superiority)
OPTION-STEMI Trial, 2025 [58]	994 (100%)	Immediate complete revascularization (*n* = 498)	Staged complete revascularization during index admission (*n* = 496)	Angiography, ≥70% or 50–69% with FFR ≤ 0.80	All-cause death, nonfatal MI, unplanned revascularization at 1-year follow-up	13.0% vs. 11.0%; HR: 1.24 (95% CI: 0.86–1.79, *p* = 0.24 for non-inferiority)

ACS: acute coronary syndrome, CI: confidence interval, CS: cardiogenic shock, CV: cardiovascular, FFR: fractional flow reserve, FU: follow-up, HF: heart failure, HR: hazard ratio, iFR: instantaneous wave-free ratio, MACE: major adverse cardiovascular events, MI: myocardial infarction, RR: risk ratio.

## Data Availability

All data underlying this article will be shared on reasonable request to the corresponding author.

## Abbreviations

The following abbreviations are used in this manuscript:

ACS	Acute Coronary Syndrome
CABG	Coronary Artery Bypass Grafting
CAD	Coronary Artery Disease
CCS	Chronic Coronary Syndrome
CI	Confidence Interval
CS	Cardiogenic Shock
CV	Cardiovascular
CVD	Cardiovascular Disease
ESC	European Society of Cardiology
FFR	Fractional Flow Reserve
HF	Heart Failure
HR	Hazard Ratio
iFR	Instantaneous Wave-Free Ratio
IVUS	Intravascular Ultrasound
LAD	Left Anterior Descending Artery
LVEF	Left Ventricular Ejection Fraction
MACE	Major Adverse Cardiovascular Events
MI	Myocardial Infarction
MLA	Minimal Luminal Area
MRI	Magnetic Resonance Imaging
NIRS	Near-Infrared Spectroscopy
NSTEMI	Non-ST-Elevation Myocardial Infarction
OCT	Optical Coherence Tomography
OMT	Optimal Medical Therapy
OR	Odds Ratio
PCI	Percutaneous Coronary Intervention
QFR	Quantitative Flow Ratio
RCTs	Randomized Controlled Trials
RR	Risk Ratio
SYNTAX	SYNergy between PCI with TAXUS and Cardiac Surgery
STEMI	ST-Elevation Myocardial Infarction
TCFA	Thin-Cap Fibroatheroma

## References

[1] GBD 2017 Causes of Death Collaborators (2018). Global, Regional, and National Age-Sex-Specific Mortality for 282 Causes of Death in 195 Countries and Territories, 1980–2017: A Systematic Analysis for the Global Burden of Disease Study 2017. Lancet.

[2] Nichols M., Townsend N., Scarborough P., Rayner M. (2014). Cardiovascular Disease in Europe 2014: Epidemiological Update. Eur. Heart J..

[3] Lloyd-Jones D., Adams R.J., Brown T.M., Carnethon M., Dai S., De Simone G., Ferguson T.B., Ford E., Furie K., Gillespie C. (2010). Executive Summary: Heart Disease and Stroke Statistics—2010 Update: A Report from the American Heart Association. Circulation.

[4] Vrints C., Andreotti F., Koskinas K.C., Rossello X., Adamo M., Ainslie J., Banning A.P., Budaj A., Buechel R.R., Chiariello G.A. (2024). 2024 ESC Guidelines for the Management of Chronic Coronary Syndromes. Eur. Heart J..

[5] Byrne R.A., Rossello X., Coughlan J.J., Barbato E., Berry C., Chieffo A., Claeys M.J., Dan G.-A., Dweck M.R., Galbraith M. (2023). 2023 ESC Guidelines for the Management of Acute Coronary Syndromes. Eur. Heart J..

[6] Levine G.N., Bates E.R., Blankenship J.C., Bailey S.R., Bittl J.A., Cercek B., Chambers C.E., Ellis S.G., Guyton R.A., Hollenberg S.M. (2016). 2015 ACC/AHA/SCAI Focused Update on Primary Percutaneous Coronary Intervention for Patients with ST-Elevation Myocardial Infarction: An Update of the 2011 ACCF/AHA/SCAI Guideline for Percutaneous Coronary Intervention and the 2013 ACCF/AHA Guideline for the Management of ST-Elevation Myocardial Infarction: A Report of the American College of Cardiology/American Heart Association Task Force on Clinical Practice Guidelines and the Society for Cardiovascular Angiography and Interventions. Circulation.

[7] Keeley E.C., Boura J.A., Grines C.L. (2006). Comparison of Primary and Facilitated Percutaneous Coronary Interventions for ST-Elevation Myocardial Infarction: Quantitative Review of Randomised Trials. Lancet.

[8] Park D.-W., Clare R.M., Schulte P.J., Pieper K.S., Shaw L.K., Califf R.M., Ohman E.M., Van de Werf F., Hirji S., Harrington R.A. (2014). Extent, Location, and Clinical Significance of Non-Infarct-Related Coronary Artery Disease among Patients with ST-Elevation Myocardial Infarction. JAMA.

[9] Tripathi B., Yeh R.W., Bavishi C.P., Sardar P., Atti V., Mukherjee D., Bashir R., Abbott J.D., Giri J., Chatterjee S. (2019). Etiologies, Trends, and Predictors of Readmission in ST-Elevation Myocardial Infarction Patients Undergoing Multivessel Percutaneous Coronary Intervention. Catheter. Cardiovasc. Interv..

[10] Cui K., Lyu S., Song X., Liu H., Yuan F., Xu F., Zhang M., Wang W., Zhang M., Zhang D. (2019). Long-Term Safety and Efficacy of Staged Percutaneous Coronary Intervention for Patients with ST-Segment Elevation Myocardial Infarction and Multivessel Coronary Disease. Am. J. Cardiol..

[11] Pimor A., Auffret V., Didier R., Delaunay R., Filippi E., Hacot J.-P., Saouli D., Rouault G., Druelles P., Bot E. (2018). Immediate Complete Revascularization in Patients with ST-Segment Elevation Myocardial Infarction and Multivessel Disease Treated by Primary Percutaneous Coronary Intervention: Insights from the ORBI Registry. Arch. Cardiovasc. Dis..

[12] Widimsky P., Holmes D.R. (2011). How to Treat Patients with ST-Elevation Acute Myocardial Infarction and Multi-Vessel Disease?. Eur. Heart J..

[13] van der Schaaf R.J., Timmer J.R., Ottervanger J.P., Hoorntje J.C.A., de Boer M., Suryapranata H., Zijlstra F., Dambrink J.E. (2006). Long-term Impact of Multivessel Disease on Cause-specific Mortality after ST Elevation Myocardial Infarction Treated with Reperfusion Therapy. Heart.

[14] Forzano I., Florimonte D., Narciso V., Canonico M.E., Castiello D.S., Manzi L., Cristiano S., Spinelli A., Vallone D.M., D’Alconzo D. (2025). Optimal Medical Therapy Targeting Lipids and Inflammation for Secondary Prevention in Patients Undergoing Percutaneous Coronary Intervention. J. Clin. Med..

[15] Prati F., Romagnoli E., Gatto L., La Manna A., Burzotta F., Ozaki Y., Marco V., Boi A., Fineschi M., Fabbiocchi F. (2020). Relationship between Coronary Plaque Morphology of the Left Anterior Descending Artery and 12 Months Clinical Outcome: The CLIMA Study. Eur. Heart J..

[16] Stone G.W., Maehara A., Lansky A.J., de Bruyne B., Cristea E., Mintz G.S., Mehran R., McPherson J., Farhat N., Marso S.P. (2011). A Prospective Natural-History Study of Coronary Atherosclerosis. N. Engl. J. Med..

[17] Pinilla-Echeverri N., Mehta S.R., Wang J., Lavi S., Schampaert E., Cantor W.J., Bainey K.R., Welsh R.C., Kassam S., Mehran R. (2020). Nonculprit Lesion Plaque Morphology in Patients with ST-Segment-Elevation Myocardial Infarction: Results from the COMPLETE Trial Optical Coherence Tomography Substudys. Circ. Cardiovasc. Interv..

[18] Piccolo R., Pilgrim T., Heg D., Franzone A., Rat-Wirtzler J., Räber L., Silber S., Serruys P.W., Jüni P., Windecker S. (2015). Comparative Effectiveness and Safety of New-Generation Versus Early-Generation Drug-Eluting Stents According to Complexity of Coronary Artery Disease: A Patient-Level Pooled Analysis of 6,081 Patients. JACC Cardiovasc. Interv..

[19] Mori H., Sakurai K., Ikari Y., Fukui K., Maeda A., Akashi Y., Ako J., Ebina T., Tamura K., Namiki A. (2023). Radial versus Femoral Access in Patients Undergoing Primary Percutaneous Coronary Intervention for ST-Elevation Myocardial Infarction: A Propensity-Matched Analysis from Real-World Data of the K-ACTIVE Registry. J. Cardiol..

[20] Castiello D.S., Oliva A., Andò G., Niccoli G., Pelliccia F., Moscarella E., Montone R.A., Gragnano F., Porto I., Calabrò P. (2025). Antithrombotic Therapy in Complex Percutaneous Coronary Intervention. EuroIntervention.

[21] Stone G.W., Christiansen E.H., Ali Z.A., Andreasen L.N., Maehara A., Ahmad Y., Landmesser U., Holm N.R. (2024). Intravascular Imaging-Guided Coronary Drug-Eluting Stent Implantation: An Updated Network Meta-Analysis. Lancet.

[22] Faro D.C., Laudani C., Agnello F.G., Ammirabile N., Finocchiaro S., Legnazzi M., Mauro M.S., Mazzone P.M., Occhipinti G., Rochira C. (2023). Complete Percutaneous Coronary Revascularization in Acute Coronary Syndromes With Multivessel Coronary Disease: A Systematic Review. JACC Cardiovasc. Interv..

[23] Thiele H., Akin I., Sandri M., Fuernau G., de Waha S., Meyer-Saraei R., Nordbeck P., Geisler T., Landmesser U., Skurk C. (2017). PCI Strategies in Patients with Acute Myocardial Infarction and Cardiogenic Shock. N. Engl. J. Med..

[24] Bangalore S., Mancini G.B.J., Leipsic J., Budoff M.J., Xu Y., Anthopolos R., Brilakis E.S., Dwivedi A., Spertus J.A., Jones P.G. (2025). Invasive vs Conservative Management of Patients With Chronic Total Occlusion: Results From the ISCHEMIA Trial. J. Am. Coll. Cardiol..

[25] Paradies V., Waldeyer C., Laforgia P.L., Clemmensen P., Smits P.C. (2021). Completeness of Revascularisation in Acute Coronary Syndrome Patients with Multivessel Disease. EuroIntervention.

[26] Hanratty C.G., Koyama Y., Rasmussen H.H., Nelson G.I.C., Hansen P.S., Ward M.R. (2002). Exaggeration of Nonculprit Stenosis Severity during Acute Myocardial Infarction: Implications for Immediate Multivessel Revascularization. J. Am. Coll. Cardiol..

[27] Politi L., Sgura F., Rossi R., Monopoli D., Guerri E., Leuzzi C., Bursi F., Sangiorgi G.M., Modena M.G. (2010). A Randomised Trial of Target-Vessel versus Multi-Vessel Revascularisation in ST-Elevation Myocardial Infarction: Major Adverse Cardiac Events during Long-Term Follow-Up. Heart.

[28] Wald D.S., Morris J.K., Wald N.J., Chase A.J., Edwards R.J., Hughes L.O., Berry C., Oldroyd K.G. (2013). PRAMI Investigators Randomized Trial of Preventive Angioplasty in Myocardial Infarction. N. Engl. J. Med..

[29] Gershlick A.H., Khan J.N., Kelly D.J., Greenwood J.P., Sasikaran T., Curzen N., Blackman D.J., Dalby M., Fairbrother K.L., Banya W. (2015). Randomized Trial of Complete versus Lesion-Only Revascularization in Patients Undergoing Primary Percutaneous Coronary Intervention for STEMI and Multivessel Disease: The CvLPRIT Trial. J. Am. Coll. Cardiol..

[30] Mehta S.R., Wood D.A., Storey R.F., Mehran R., Bainey K.R., Nguyen H., Meeks B., Di Pasquale G., López-Sendón J., Faxon D.P. (2019). Complete Revascularization with Multivessel PCI for Myocardial Infarction. N. Engl. J. Med..

[31] Mehta S.R., Tiong D.T.W., Böhm F., Ramasundarahettige C., Biscaglia S., Campo G., James S., Smits P.C., Giacoppo D., McCann G.P. (2025). Complete versus Culprit Lesion-Only Revascularisation for Acute Myocardial Infarction (Complete Revascularisation Trialists’ Collaboration): An Individual Patient Data Meta-Analysis of Randomised Trials. Lancet.

[32] van der Hoeven N.W., Janssens G.N., de Waard G.A., Everaars H., Broyd C.J., Beijnink C.W.H., van de Ven P.M., Nijveldt R., Cook C.M., Petraco R. (2019). Temporal Changes in Coronary Hyperemic and Resting Hemodynamic Indices in Nonculprit Vessels of Patients With ST-Segment Elevation Myocardial Infarction. JAMA Cardiol..

[33] Engstrøm T., Kelbæk H., Helqvist S., Høfsten D.E., Kløvgaard L., Holmvang L., Jørgensen E., Pedersen F., Saunamäki K., Clemmensen P. (2015). Complete Revascularisation versus Treatment of the Culprit Lesion Only in Patients with ST-Segment Elevation Myocardial Infarction and Multivessel Disease (DANAMI-3—PRIMULTI): An Open-Label, Randomised Controlled Trial. Lancet.

[34] Smits P.C., Abdel-Wahab M., Neumann F.-J., Boxma-de Klerk B.M., Lunde K., Schotborgh C.E., Piroth Z., Horak D., Wlodarczak A., Ong P.J. (2017). Fractional Flow Reserve-Guided Multivessel Angioplasty in Myocardial Infarction. N. Engl. J. Med..

[35] Puymirat E., Cayla G., Simon T., Steg P.G., Montalescot G., Durand-Zaleski I., le Bras A., Gallet R., Khalife K., Morelle J.-F. (2021). Multivessel PCI Guided by FFR or Angiography for Myocardial Infarction. N. Engl. J. Med..

[36] Lee J.M., Kim H.K., Park K.H., Choo E.H., Kim C.J., Lee S.H., Kim M.C., Hong Y.J., Ahn S.G., Doh J.-H. (2023). Fractional Flow Reserve versus Angiography-Guided Strategy in Acute Myocardial Infarction with Multivessel Disease: A Randomized Trial. Eur. Heart J..

[37] Böhm F., Mogensen B., Engstrøm T., Stankovic G., Srdanovic I., Lønborg J., Zwackman S., Hamid M., Kellerth T., Lauermann J. (2024). FFR-Guided Complete or Culprit-Only PCI in Patients with Myocardial Infarction. N. Engl. J. Med..

[38] Biscaglia S., Guiducci V., Escaned J., Moreno R., Lanzilotti V., Santarelli A., Cerrato E., Sacchetta G., Jurado-Roman A., Menozzi A. (2023). Complete or Culprit-Only PCI in Older Patients with Myocardial Infarction. N. Engl. J. Med..

[39] Götberg M., Christiansen E.H., Gudmundsdottir I.J., Sandhall L., Danielewicz M., Jakobsen L., Olsson S.-E., Öhagen P., Olsson H., Omerovic E. (2017). Instantaneous Wave-Free Ratio versus Fractional Flow Reserve to Guide PCI. N. Engl. J. Med..

[40] Nijveldt R., Maeng M., Beijnink C.W.H., Piek J.J., Al-Lamee R.K., Raposo L., Baptista S.B., Escaned J., Davies J., Klem I. (2026). Immediate or Deferred Nonculprit-Lesion PCI in Myocardial Infarction. N. Engl. J. Med..

[41] Elendu C., Amaechi D.C., Elendu T.C., Omeludike E.K., Alakwe-Ojimba C.E., Obidigbo B., Akpovona O.L., Oros Sucari Y.P., Saggi S.K., Dang K. (2023). Comprehensive Review of ST-Segment Elevation Myocardial Infarction: Understanding Pathophysiology, Diagnostic Strategies, and Current Treatment Approaches. Medicine.

[42] Vergallo R., Porto I., D’Amario D., Annibali G., Galli M., Benenati S., Bendandi F., Migliaro S., Fracassi F., Aurigemma C. (2019). Coronary Atherosclerotic Phenotype and Plaque Healing in Patients With Recurrent Acute Coronary Syndromes Compared With Patients With Long-Term Clinical Stability: An In Vivo Optical Coherence Tomography Study. JAMA Cardiol..

[43] Räber L., Mintz G.S., Koskinas K.C., Johnson T.W., Holm N.R., Onuma Y., Radu M.D., Joner M., Yu B., Jia H. (2018). Clinical Use of Intracoronary Imaging. Part 1: Guidance and Optimization of Coronary Interventions. An Expert Consensus Document of the European Association of Percutaneous Cardiovascular Interventions. Eur. Heart J..

[44] Johnson T.W., Räber L., di Mario C., Bourantas C., Jia H., Mattesini A., Gonzalo N., de la Torre Hernandez J.M., Prati F., Koskinas K. (2019). Clinical Use of Intracoronary Imaging. Part 2: Acute Coronary Syndromes, Ambiguous Coronary Angiography Findings, and Guiding Interventional Decision-Making: An Expert Consensus Document of the European Association of Percutaneous Cardiovascular Interventions. Eur. Heart J..

[45] Karamasis G.V., Varlamos C., Benetou D.-R., Kalogeropoulos A.S., Keeble T.R., Tsigkas G., Xenogiannis I. (2023). The Usefulness of Intracoronary Imaging in Patients with ST-Segment Elevation Myocardial Infarction. J. Clin. Med..

[46] Kedhi E., Berta B., Roleder T., Hermanides R.S., Fabris E., IJsselmuiden A.J.J., Kauer F., Alfonso F., von Birgelen C., Escaned J. (2021). Thin-Cap Fibroatheroma Predicts Clinical Events in Diabetic Patients with Normal Fractional Flow Reserve: The COMBINE OCT-FFR Trial. Eur. Heart J..

[47] Erlinge D., Maehara A., Ben-Yehuda O., Bøtker H.E., Maeng M., Kjøller-Hansen L., Engstrøm T., Matsumura M., Crowley A., Dressler O. (2021). Identification of Vulnerable Plaques and Patients by Intracoronary Near-Infrared Spectroscopy and Ultrasound (PROSPECT II): A Prospective Natural History Study. Lancet.

[48] Xu X., Fang C., Jiang S., Chen Y., Zhao J., Sun S., Wang Y., Li L., Huang D., Li S. (2025). Functional or Anatomical Assessment of Non-Culprit Lesions in Acute Myocardial Infarction. EuroIntervention.

[49] Kim H., Ahn J.-M., Kang D.-Y., Lee J., Choi Y., Park S.-J., Park D.-W. (2024). Management of Coronary Vulnerable Plaque With Medical Therapy or Local Preventive Percutaneous Coronary Intervention. JACC Asia.

[50] Forzano I., Narciso V., Canonico M.E., Castiello D.S., Florimonte D., Manzi L., Semplice F., Vallone D.M., Cristiano S., Spinelli A. (2026). Optimal Medical Therapy Targeting Metabolic Status for Secondary Prevention in Patients Undergoing Percutaneous Coronary Intervention. J. Clin. Med..

[51] Zorina O., Fatkulina N., Saduyeva F., Omarkulov B., Serikova S. (2022). Patient Adherence to Therapy After Myocardial Infarction: A Scoping Review. Patient Prefer. Adherence.

[52] Park S.-J., Ahn J.-M., Kang D.-Y., Yun S.-C., Ahn Y.-K., Kim W.-J., Nam C.-W., Jeong J.-O., Chae I.-H., Shiomi H. (2024). Preventive Percutaneous Coronary Intervention versus Optimal Medical Therapy Alone for the Treatment of Vulnerable Atherosclerotic Coronary Plaques (PREVENT): A Multicentre, Open-Label, Randomised Controlled Trial. Lancet.

[53] Harskamp R.E., Bonatti J.O., Zhao D.X., Puskas J.D., de Winter R.J., Alexander J.H., Halkos M.E. (2014). Standardizing Definitions for Hybrid Coronary Revascularization. J. Thorac. Cardiovasc. Surg..

[54] Kastrati A., Kessler T., Rinaldi R., Brugaletta S. (2024). Complete Revascularisation Should Be Immediate in STEMI: Pros and Cons. EuroIntervention.

[55] Wood D.A., Cairns J.A., Wang J., Mehran R., Storey R.F., Nguyen H., Meeks B., Kunadian V., Tanguay J.-F., Kim H.-H. (2019). Timing of Staged Nonculprit Artery Revascularization in Patients With ST-Segment Elevation Myocardial Infarction: COMPLETE Trial. J. Am. Coll. Cardiol..

[56] Diletti R., den Dekker W.K., Bennett J., Schotborgh C.E., van der Schaaf R., Sabaté M., Moreno R., Ameloot K., van Bommel R., Forlani D. (2023). Immediate versus Staged Complete Revascularisation in Patients Presenting with Acute Coronary Syndrome and Multivessel Coronary Disease (BIOVASC): A Prospective, Open-Label, Non-Inferiority, Randomised Trial. Lancet.

[57] Stähli B.E., Varbella F., Linke A., Schwarz B., Felix S.B., Seiffert M., Kesterke R., Nordbeck P., Witzenbichler B., Lang I.M. (2023). Timing of Complete Revascularization with Multivessel PCI for Myocardial Infarction. N. Engl. J. Med..

[58] Kim M.C., Ahn J.H., Hyun D.Y., Lim Y., Cho K.H., Lee S.H., Park S., Oh S., Sim D.S., Hong Y.J. (2025). Immediate versus Staged Complete Revascularisation during Index Admission in Patients with ST-Segment Elevation Myocardial Infarction and Multivessel Disease (OPTION-STEMI): A Multicentre, Non-Inferiority, Open-Label, Randomised Trial. Lancet.

[59] Elbahloul M.A., Ramadan S., Labeeb E.E., Aldalahmeh S., Mousa A.I.A., Elazab A., Mohamed A.N., Elashery A., El-Hamdani M.O., Lavie C.J. (2026). Timing of Complete Revascularization in Patients With STEMI and Multivessel Disease: An Updated Meta-Analysis of Randomized Clinical Trials. Circ. Cardiovasc. Interv..

[60] Thiele H., Ohman E.M., de Waha-Thiele S., Zeymer U., Desch S. (2019). Management of Cardiogenic Shock Complicating Myocardial Infarction: An Update 2019. Eur. Heart J..

[61] Osman M., Syed M., Patibandla S., Sulaiman S., Kheiri B., Shah M.K., Bianco C., Balla S., Patel B. (2021). Fifteen-Year Trends in Incidence of Cardiogenic Shock Hospitalization and In-Hospital Mortality in the United States. J. Am. Heart Assoc..

[62] Webb J.G., Lowe A.M., Sanborn T.A., White H.D., Sleeper L.A., Carere R.G., Buller C.E., Wong S.C., Boland J., Dzavik V. (2003). Percutaneous Coronary Intervention for Cardiogenic Shock in the SHOCK Trial. J. Am. Coll. Cardiol..

[63] Hochman J.S., Sleeper L.A., Webb J.G., Sanborn T.A., White H.D., Talley J.D., Buller C.E., Jacobs A.K., Slater J.N., Col J. (1999). Early Revascularization in Acute Myocardial Infarction Complicated by Cardiogenic Shock. SHOCK Investigators. Should We Emergently Revascularize Occluded Coronaries for Cardiogenic Shock. N. Engl. J. Med..

[64] Møller J.E., Engstrøm T., Jensen L.O., Eiskjær H., Mangner N., Polzin A., Schulze P.C., Skurk C., Nordbeck P., Clemmensen P. (2024). Microaxial Flow Pump or Standard Care in Infarct-Related Cardiogenic Shock. N. Engl. J. Med..

[65] Marquard J.M., Beske R.P., Hassager C., Jensen L.O., Eiskjær H., Mangner N., Polzin A., Schulze C., Skurk C., Nordbeck P. (2025). Percutaneous Coronary Intervention in Multivessel Disease and Infarct-Related Cardiogenic Shock: A DanGer Shock Substudy. JACC Cardiovasc. Interv..

[66] Angiolillo D.J., Galli M., Collet J.-P., Kastrati A., O’Donoghue M.L. (2022). Antiplatelet Therapy after Percutaneous Coronary Intervention. EuroIntervention.

[67] Oliva A., Castiello D.S., Franzone A., Condorelli G., Colombo A., Esposito G., Stefanini G.G., Piccolo R. (2023). P2Y12 Inhibitors Monotherapy in Patients Undergoing Complex vs Non-Complex Percutaneous Coronary Intervention: A Meta-Analysis of Randomized Trials. Am. Heart J..

[68] Gargiulo G., Cirillo P., Sperandeo L., Castiello D.S., Manzi L., Forzano I., Florimonte D., Simonetti F., Canonico M.E., Avvedimento M. (2025). Pharmacodynamic Effects of Cangrelor in Patients with Acute or Chronic Coronary Syndrome Undergoing Percutaneous Coronary Intervention: The POMPEII Registry. EuroIntervention.

[69] Gargiulo G., Narciso V., Forzano I., Sperandeo L., Castiello D.S., Simonetti F., Manzi L., Florimonte D., Canonico M.E., Avvedimento M. (2026). Pharmacodynamic Effects of Cangrelor in Patients with or without STEMI Undergoing Percutaneous Coronary Intervention: Insights from the POMPEII Study. Eur. Heart J. Acute Cardiovasc. Care.

[70] Emilsson O.L., Mohammad M.A., Grimfjärd P., Persson J., Santos-Pardo I., Erlinge D., Koul S. (2025). Cangrelor During Percutaneous Coronary Intervention in Patients With Cardiogenic Shock or Cardiac Arrest. JACC Cardiovasc. Interv..

[71] Giustino G., Chieffo A., Palmerini T., Valgimigli M., Feres F., Abizaid A., Costa R.A., Hong M.-K., Kim B.-K., Jang Y. (2016). Efficacy and Safety of Dual Antiplatelet Therapy After Complex PCI. J. Am. Coll. Cardiol..

[72] Tian J., Wang Z., Wang Y., Wang F., Wang Y., Zhao P., Hou X., Peng X., Tian M., Wang D. (2024). Rationale and Design of Dual Antiplatelet Therapy in Patients with Coronary Multi-Vessel Disease (DAPT-MVD): A Multicenter, Randomized, Controlled Trial. Clin. Cardiol..

[73] Piccolo R., Calabrò P., Carrara G., Simonetti F., Varricchio A., Attisano T., Napolitano G., De Simone C., Carpinella G., Stabile E. (2025). Personalized or Standard Duration of Dual Antiplatelet Therapy After Percutaneous Coronary Intervention: The PARTHENOPE Randomized Trial. J. Am. Coll. Cardiol..

[74] Castiello D.S., Buongiorno F., Manzi L., Narciso V., Forzano I., Florimonte D., Sperandeo L., Canonico M.E., Avvedimento M., Paolillo R. (2025). Procedural and Antithrombotic Therapy Optimization in Patients with Atrial Fibrillation Undergoing Percutaneous Coronary Intervention: A Narrative Review. J. Cardiovasc. Dev. Dis..

[75] Erriquez A., Colaiori I., Hakeem A., Guiducci V., Menozzi M., Barbierato M., Arioti M., D’Amario D., Casella G., Scarsini R. (2025). Functional Coronary Angiography to Indicate and Guide Revascularization in STEMI Patients with Multivessel Disease: Rationale and Design of the AIR-STEMI Trial. Am. Heart J..

[76] Iannaccone M., DE Filippo O., Montabone A., Marengo G., Maltese L., Ugo F., Quadri G., Mennuni M., Secco G.G., Taglialatela V. (2023). OCT Guided vs. COmplete Pci in patieNts with sT Segment Elevation myocArdial infarCtion and mulTivessel Disease: OCT-CONTACT RCT. Minerva Cardiol. Angiol..

[77] Chen X., Li S., Wang B., Chen G., Yao Y., He Y., Wu X., Yang Y., Kang H., Ding L. (2026). Staged Interventional Strategies for Acute ST-Segment Elevation Myocardial Infarction Patient with Multivessel Disease: Rationale and Design of a Multicenter, Randomized STAGED Trial. Am. Heart J..

